# Self-Induced Myofascial Release in Patients with Hemophilic Ankle Arthropathy: A Pilot Observational Study

**DOI:** 10.3390/life12122008

**Published:** 2022-12-02

**Authors:** Elena Donoso-Úbeda, Raúl Pérez-Llanes, Javier Meroño-Gallut, Roberto Ucero-Lozano, Rubén Cuesta-Barriuso

**Affiliations:** 1 Department of Physiotherapy, Catholic University San Antonio-UCAM, 30107 Guadalupe, Spain; 2 Physiotherapy Service, Tu Bienestar 360°, 30730 San Javier, Spain; 3 Department of Physiotherapy, Faculty of Sport Sciences, European University of Madrid, 28670 Madrid, Spain; 4 Department of Surgery and Medical-Surgical Specialties, University of Oviedo, 33006 Asturias, Spain; 5 Royal Victoria Eugenia Foundation, 28029 Madrid, Spain

**Keywords:** hemophilia, ankle arthropathy, self-induced myofascial release, manual therapy, frequency of hemarthrosis

## Abstract

Background: Hemophilic ankle arthropathy is manifested by degenerative functional alterations (reduced muscle strength, mobility, and proprioception) and chronic pain. Myofascial release techniques are used to treat soft tissue adhesions, relieve pain, and reduce tissue sensitivity. The aim was to evaluate the safety of self-induced myofascial release in patients with hemophilic ankle arthropathy and to assess possible changes in musculoskeletal variables. Methods: We recruited 20 patients with ankle hemophilic arthropathy. Patients carried out a daily self-induced myofascial release exercise program using a foam roller over a period of 8 weeks. The primary variable was the frequency of hemarthrosis (regular telephone follow-up). Secondary variables were pain intensity (visual analog scale), range of motion (goniometry), and functional capacity of the lower limbs (six-minute walk test). Three evaluations were performed: pre-treatment (T0), post-treatment (T1), and at 8 weeks follow-up (T2). Results: There was a lower, non-significant, association in the frequency of hemarthrosis between the experimental and follow-up periods, compared to the pre-study period (SE = 0.50; 95%CI: −1.67; 0.28). There were significant within-subject changes in intensity of pain (T0: 4.91; T1: 2.79; T2: 2.46; *p* < 0.001), plantar flexion (T0: 125.55; T1: 131.5; T2: 130.30; *p* = 0.01), and functionality of the lower limbs (T0: 173.06; T1: 184.85; T2: 178.39; *p* = 0.009). Conclusions: Self-induced myofascial release is safe in patients with hemophilic ankle arthropathy. A protocol based on self-induced myofascial release can lead to changes in pain intensity, range of ankle motion in plantar flexion, and functionality in hemophilic patients.

## 1. Introduction

Hemophilia is an X-linked clotting disorder characterized by the deficiency or absence of certain clotting factors. Hemophilia A is characterized by a deficiency of clotting factor VIII, while factor IX is missing in hemophilia B [1]. Depending on plasma clotting factor levels, hemophilia can be classified as severe (<1%), moderate (1–5%), and mild (5–40%) [2].

The main clinical manifestation of hemophilia is bleeding in the locomotor system (especially in muscles and joints). The recurrence of intra-articular hemorrhages (hemarthrosis) leads to a degenerative, chronic, and progressive process (hemophilic arthropathy). The development of a chronic inflammatory process, known as chronic synovial hypertrophy, is common in patients with hemophilic arthropathy. Arthropathy is characterized by chronic pain, reduced mobility [1,3], axial alterations, chronic synovitis and hypertrophy of the epiphyseal plate [4].

Ankles, as well as knees and elbows, are the joints that most exhibit hemarthrosis in these patients [5]. The first bleeds occur when the child with hemophilia begins to crawl and walk. In the second decade of life, the high incidence of hemarthrosis in this population leads to the development of hemophilic arthropathy [6]. Prophylactic treatment with clotting factor concentrates is the most effective strategy to prevent or minimize the development of hemarthrosis and therefore hemophilic arthropathy [7].

Chronic ankle pain greatly influences the patient’s perceived quality of life. However, identifying pain and its origin is not always accurate, hence treatment for its control may not be suitable [8]. Various physiotherapy techniques, such as proprioception, strength and stability training at home, or manual therapy using joint traction and passive stretching, have been shown to improve the perception of pain in patients with hemophilic ankle arthropathy [9,10,11].

Fascial therapy aims to reduce perceived pain while improving functionality and proprioception. This technique is based on mobilization of connective tissue by applying gentle, sustained pressure [12]. The safety of this technique for people with hemophilia has been widely demonstrated [13,14,15,16,17,18]. Self-induced myofascial release is based on the same principles as fascial therapy. However, the pressure exerted in this technique is produced by the weight of the patient’s body on a foam roller [19]. It should be borne in mind that the effects of manual therapy on pain perception involve a number of factors in addition to those directly related to the intervention. The patient’s expectations, the patient-physiotherapist relationship in the clinical encounter, the characteristics of the treatment environment, or the analgesic capacity of touch are influential elements in the reduction of pain or the regulation of the patient’s emotions [20,21,22].

Self-induced myofascial release is used to reduce fascial adhesions, improving mobility between the various layers of connective tissue [23]. The thixotropic property of the fascia is responsible for facilitating this therapeutic process [19]. Foam roller-based self-induced fascial therapy has been shown to be effective in improving the range of ankle motion and perceived pain in patients with plantar fasciitis [24]. The use of a foam roller using only the pressure of the body’s weight without actually rolling, has shown its effectiveness in reducing pressure pain in latent myofascial trigger points in the lateral gastrocnemius muscle in healthy subjects [25]. On the other hand, its use in athletes diagnosed with mild distension or decreased strength and flexibility in the hamstrings muscle group has shown its effectiveness in improving the flexibility of this muscle group [26].

The alternative study hypothesis is that a myofascial self-release intervention is safe in terms of the frequency of hemarthrosis, measured by periodic monitoring, for patients with hemophilia and ankle arthropathy. The main objective of the study was to evaluate the safety of a home self-myofascial release program by evaluating the frequency of joint bleeding in adult people with hemophilic ankle arthropathy. The secondary objective was to evaluate any changes in pain intensity, ankle range of motion, and lower limb functionality.

## 2. Materials and Methods

### 2.1. Study Design

A multicenter, single-blind observational pilot study to assess the safety of self-induced myofascial release in adult patients with hemophilic ankle arthropathy. The patients were recruited by the Spanish Federation of Hemophilia and the Malaga Association of Hemophilia between September and November 2020.

### 2.2. Local Approvals

One of the study researchers informed the patient associations about the selection criteria and goals set for the study. After gathering the potential participants, they were informed of the study’s risks and benefits, providing an information sheet with the most relevant data. All participants signed the Informed Consent Document. Patients who met the selection criteria were informed verbally and in writing of the objectives, risks, and benefits of the intervention. All subjects signed the informed consent document, in accordance with the Helsinki statement. This study was approved by the Research Ethics Committee of the University of Murcia (ID: 2428/2019). The research project was registered prospectively with the International Clinical Trials Registry (www.clinicaltrials.gov, accessed on 16 April 2019; NCT03914287).

### 2.3. Participants

The sample size was calculated using the statistical package G * Power (version 3.1.9.2; Heinrich-Heine-Universität Düsseldorf, Düsseldorf, Germany). Assuming a mean effect size (d = 0.60), with an alpha level (type I error) of 0.05 and a statistical power of 90% (1 − β = 0.90), a sample size of 17 patients with hemophilic ankle arthropathy was estimated. Estimating a 20% percentage of dropouts or non-compliance with the protocol, we estimated the inclusion of 20 patients with hemophilia. The patients were recruited from two Spanish regions (Madrid and Malaga) through the Spanish Federation of Hemophilia and the Malaga Association of Hemophilia.

Inclusion criteria were: being over 18 years old; having a medical diagnosis of hemophilia A or B; having no scheduled orthopedic surgeries during the study phase; signing the informed consent document; and having a medical diagnosis of hemophilic ankle arthropathy and more than 3 points on the Hemophilia Joint Health Score. This scale [27] assesses nine variables: inflammation, atrophy and muscle strength, crepitus, mobility and joint pain, and gait. The results of this scale express the degree of joint deterioration in patients with hemophilia ranging from 0 to 20 (maximum joint damage) points.

The exclusion criteria were: patients with ankle hemarthrosis in the month before the beginning of the study; patients who were unable to walk even with technical aids; patients with hemophilic elbow arthropathy that prevented the performance of the exercises; and patients who failed to complete at least 80% of the sessions scheduled in the intervention.

All patients continued to follow the therapeutic regimen prescribed by their hematologist. Inclusion in the study did not require any modification of the doses or days of administration of FVIII/FIX concentrates. Twenty-five adult patients with hemophilic ankle arthropathy were invited to participate in this study. Three patients were excluded due to knee (2) and ankle (1) hemarthrosis in the previous month. One patient could not be included because of an ankle synoviorthesis scheduled during the study phase, and another declined to participate. Finally, 20 patients with hemophilic ankle arthropathy were included in the study.

### 2.4. Outcome Measures

Three evaluations were performed in this study: T0 (at baseline), T1 (at the end of the 8-week experimental phase), and T2 (after an 8-week follow-up). All evaluations were performed by the same evaluator reproducing the same protocol and under the same conditions. The evaluator, 45 years old and with more than 20 years of clinical experience in the musculoskeletal evaluation of patients, was blinded regarding the study conditions and objectives. Before starting the intervention, the main independent clinical variables (type and severity of hemophilia, type of treatment, development of inhibitors, and ankle joint health), anthropometric variables (weight and height), and sociodemographic variables (age) were collected. The primary variable of the study to measure the safety of the intervention was the frequency of ankle hemarthrosis. The secondary variables were pain intensity, range of motion, and lower-limb functionality.

The safety of the intervention was evaluated through periodic monitoring of the frequency of ankle hemarthrosis. The physiotherapist in charge of evaluating the patients performed weekly follow-up telephone calls during the experimental phase to evaluate the development of ankle hemarthrosis or other complications (bruising). The rater had a questionnaire with closed-ended questions to be completed with closed-ended answers. These questions referred to the main clinical manifestations of hemarthrosis: pain, functional disability, swelling, heat, etc. Bleeding-related data were collected by the evaluator after the follow-up period. This evaluation of the frequency of hemarthrosis has already been carried out in previous studies to verify the safety of different physiotherapy techniques in patients with hemophilia [28,29].

The intensity of joint pain was assessed using the visual analog scale (VAS) [30]. This scale has shown high test-retest reliability (ICC: 0.99) [31]. This scale rates ankle joint pain with scores from zero to 10 points (from no pain to the maximum perceived pain).

The range of ankle motion in dorsal and plantar flexion was measured with a universal goniometer in one-degree increments. This measuring instrument has shown good intra-rater reliability (ICC: 0.85–0.96) [32]. The patient was placed in a supine position, with the axis of the goniometer on the lateral malleolus and the fixed arm of the goniometer parallel to the fibula. This procedure was accompanied by verbal stimulus to the patient to control for the compensatory movement of the toes and the range of movement of the talocrural joint [33]. The unit of measurement is the degree (the higher the mark, the better the range of motion).

Functional capacity was measured with the 2-Minute Walk test, using the standardization described by the American Thoracic Society [34]. The test was performed on a flat, hard, straight, 20-m long surface. Before the test, the evaluator instructed each patient to walk the track twice to familiarize themselves with the test and warm up. The evaluating physiotherapist used standardized verbal stimuli during the test with all subjects. Patients were asked to walk at a constant speed for two minutes without jumping or running. To measure the exact distance walked during the test, the physiotherapist closely followed the patients with a stopwatch.

### 2.5. Intervention

The physiotherapist, not being the same therapist as the evaluator, explained the main characteristics of the intervention individually to each patient. During this first face-to-face session, each exercise was carefully explained, and relevant adaptations were made according to the limitations of each patient for the proper execution of the exercises. The exercises under the specific protocol for patients with hemophilic arthropathy were [35]:

- Self-release of the plantar region of the foot with a foam ball. The patient sat in a chair, adjusting foot pressure with his or her own weight. The patient performed five circular slides over the region near the heel, the middle plantar region, and the metatarsal head. Likewise, 15 longitudinal slides occur from the heel to the metatarsal head while simultaneously flexing-extending the toes. Both exercises were conducted bilaterally.
- Release of the posterior region of the leg with the foam roller. With the patient sitting on the floor, the foam roller was placed at the most distal region of the leg. Fifteen slow slides, reaching the area of the myotendinous junction of the calf muscles, and then over the region of the soleus and gastrocnemius muscles. The patient assisted by actively performing flexion-extension movements of the ankle. Optionally, the patient could complete the exercises by crossing the contralateral leg in extension over the treated leg.
- Release of the anterior leg region with a foam roller. In a quadruped position, the patient places one leg on the roller, resting on the anterior region of the ankle. Fifteen slow movements were made by exerting a slight pressure on the roller. The patient was allowed to flex and extend the ankle. In addition, in the same position with slight hip rotation, the patient performed 15 slow movements on the roller with the anterolateral region of the leg.

**Figure 1:**
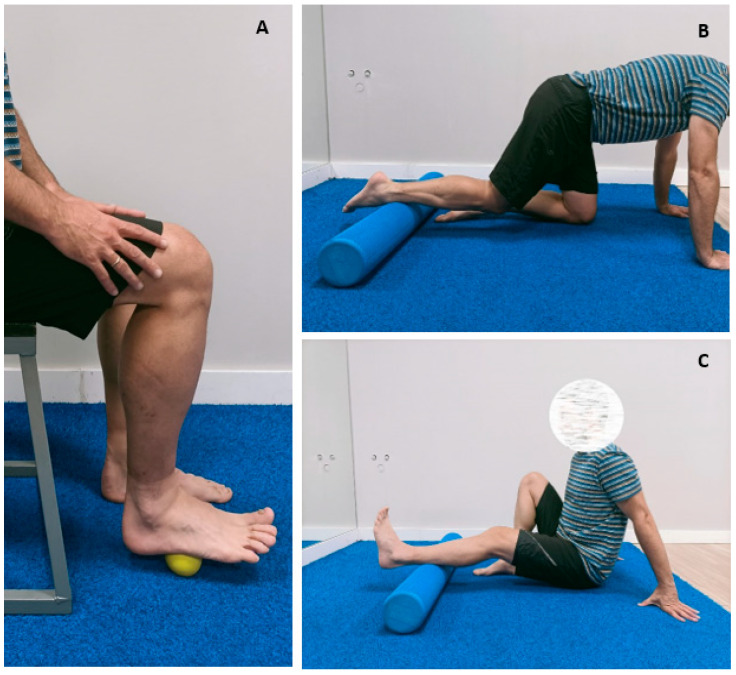
Exercises developed during the study. (**A**) Self-release on the sole of the foot; (**B**) Release of the ventral leg region; (**C**) Release of the dorsal leg region.

At home, the patients performed a daily session, lasting 15 min each, over 8 weeks. The material used was a foam roller 30 cm in length and 15 cm in diameter and a foam ball 8 cm in diameter. Patients accessed an ad hoc mobile application designed by the Hemophilia Physiotherapy research group (He-Foam^®^). This app, accessible from any smartphone, made it possible to watch the videos with all the exercises included in the intervention. The physiotherapist responsible for the intervention carried out a weekly periodic follow-up through video calls to all patients to resolve any doubts and adapt exercises in case of pain in the upper or lower limbs, general pain, or functional limitations [35]. Figure 1 shows the exercises developed in the study.

### 2.6. Statistical Analysis

The statistical analysis was performed with the SPSS statistical package for Windows, version 19.0 (IBM Company, Armonk, NY, USA). Descriptive statistics of central tendency and dispersion of the study variables were calculated. The changes in the primary variable and frequency of hemarthrosis were analyzed using a Poisson and negative binomial regression model with time (in days) as the exposure variable. The likelihood-ratio test was calculated, estimating the dispersion parameter estimate (alpha). The analysis of the within-group effect was performed with the repeated measures ANOVA test. The error rate of the significance level was controlled using the Bonferroni correction. When Mauchly’s sphericity test was significant, the Greenhouse-Geisser correction coefficient was used. The partial Eta-squared value was used as an indicator of the effect size (classified as small 0.01, medium 0.06, and large 0.14) [36]. An intent-to-treat analysis was performed to analyze the results. The selected significance level was 0.05.

## 3. Results

### Descriptive Analysis

Twenty adult patients with hemophilia were recruited. The median age was 36 (interquartile range [IR]: 9) years. Most patients were diagnosed with hemophilia A (80%), had a severe phenotype (90%), and were on prophylactic treatment (85%). Only 15% had a history of antibodies to clotting factor concentrates (inhibitors). The average deterioration of the ankle measured on the Hemophilia Joint Health Score was 11 (IR: 6.0) points. After the study period, 12 of the 20 patients were actively following the protocol (100% compliance), and 8 patients (40%) were partially active (85.7% to 98.2% compliance). Table 1 shows the descriptive characteristics of the patients included in the study.

**Figure 2:**
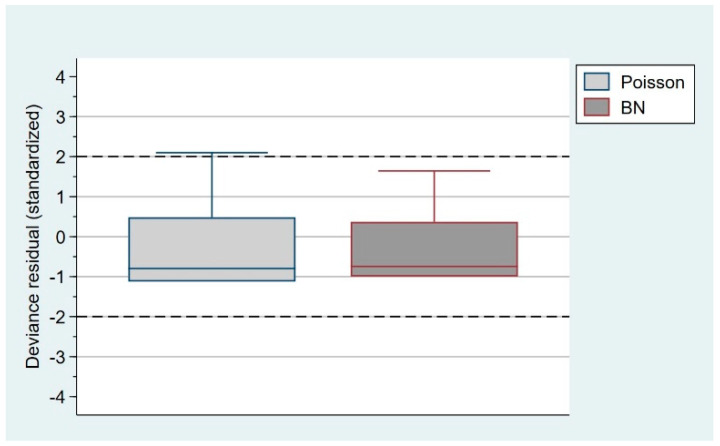
Graphical representation of the residual deviation for the regression models.

The physiotherapist in charge of adapting and monitoring the home-based self-induced myofascial release protocol followed up on patients via weekly phone calls during the 8 weeks the intervention lasted. None of the patients with hemophilia included in the study developed ankle hemarthrosis as a consequence of the intervention. Two hemarthroses were recorded during the study period and four hemarthroses during the follow-up period (secondary to trauma or spontaneous onset). When performing the Poisson regression analysis, we observed a lower association in the frequency of hemarthrosis (e^b1^ = 0.69) between the experimental phase (56 weeks) and follow-up period (56 weeks), versus the pre-study period (56 weeks). However, this association was not significant (SE = 0.50; 95% CI: −1.67–0.28). When conducting the negative binomial regression analysis, the intervention and follow-up times were associated with a lower frequency of ankle hemarthrosis (e^b1^ = 0.50). Similarly, this difference was not significant (SE = 0.51; 95% CI: −1.70–0.32). Although both models offer similar results, the dispersion parameter was estimated to select the model with the best fit. Finally, the negative binomial regression model was discarded since it failed to fit the data better than Poisson regression (χ^2^ = 0.09; *p* = 0.38). Table 2 shows the regression analysis of changes in the primary variable, the frequency of hemarthrosis. Figure 2 depicts the graphical representation of the residual deviation for the regression models used in the analysis.

In the repeated measures analysis, there were within-group changes (*p* < 0.01) in the variables: pain intensity (F (2) = 33.68; η^2^_p_ = 0.63), dorsal flexion (F (27.76) = 15.87; η^2^_p_ = 0.45) and plantar flexion (F (2) = 7.10; η^2^_p_ = 0.27) of the ankle, and lower limb functionality (F (19.01) = 5.34; η^2^_p_ = 0.22). Table 3 shows the results of the repeated measures analysis.

The pairwise comparison analysis exhibited significant changes (*p* < 0.05) between T0–T1 assessments in all dependent variables, except for dorsal flexion (*p* = 0.57). There were no differences between post-treatment and follow-up evaluations (*p* > 0.05). After the follow-up period, when comparing the results with the baseline assessments (T0–T2), only small changes were found (*p* < 0.05) in the intensity of ankle pain. Table 4 shows the results of the pairwise comparison analysis.

## 4. Discussion

The main objective of this study was to evaluate the safety of a home-based self-induced myofascial release program using a foam roller in patients with hemophilic ankle arthropathy. During the intervention, two patients developed ankle hemarthrosis due to trauma. During the follow-up phase, four ankle bleeds were observed.

The safety of self-induced myofascial release, in terms of the incidence of hemarthrosis, is consistent with the results obtained after applying manual myofascial therapy techniques in people with hemophilic ankle arthropathy [13,14,15,28]. The tension in the periarticular fascial system may decrease after a foam roller-based exercise protocol. This decrease may reduce the mechanical joint pressure typically present in hemophilic arthropathy, thus favoring a lower frequency of hemarthrosis. Such techniques are based on applying sustained pressure and slow, non-aggressive movements. Implementing the same without reaching the maximum limits of joint range of motion avoids the risk component posed by intra-articular tension with maximum joint range [37].

Chronic pain is one of the variables that most importantly leads to loss of function and impacts on the perceived quality of life in patients with hemophilic arthropathy [38]. The potential effectiveness of manual therapy, through the peripheral nervous system, in reducing musculoskeletal pain has been suggested in the scientific literature [39]. After the experimental phase, there were changes in the intensity of joint pain with a large effect size. Although, due to the study design, we are unable to assert a cause-and-effect relationship between the intervention and the outcome, the changes observed are consistent with those reported by Aishwarya et al. [24] in terms of a reduction in ankle pain in people with plantar fasciitis. Increased blood flow caused by the foam roller intervention may contribute to the elimination of waste products, thus facilitating the reduction of pain [24]. Changes in the levels of different chemicals (a decrease in cortisol and an increase in dopamine and serotonin) derived from interventions on the soft tissues are due to activation of the parasympathetic nervous system [24,40]. Finally, the lowered pain intensity after myofascial self-release using a foam roller may be favored by the restoration of tissue extensibility [40].

Between the different evaluations, we observed changes in the range of motion in plantar flexion, although these changes disappeared after the follow-up period. These changes have also been identified in adolescent athletes who followed a 6-month myofascial self-release protocol using a foam roller [41]. Applying pressure using the patient’s own body can favor a change in fascial tissue density. The thixotropic property of the fascia can modify its density, thereby increasing the elasticity of tissues [19]. Studies in animal models have established that myofascial release can improve inflammatory processes by decreasing proinflammatory mediators (NF-kB, IL-1β, TNF-α and COX-2) and increasing anti-inflammatory mediators (PPAR-γ) [42]. Although our results cannot be considered effective due to the intervention design, they point to a possible clinical improvement that may be relevant in these patients with severe range of motion limitations.

At the end of the intervention, a significant improvement was observed in the functional capacity of the patients included in the study. This improvement is consistent with the changes obtained after fascial therapy in people with hemophilic ankle arthropathy [28]. These findings suggest that there is a direct relationship between the perceived improvement in the perception of pain and in ankle range of motion and the changes experienced with the increased functional activity of the joint.

The results of this study have been obtained taking only the direct effect of the intervention into account. Ideally, the changes in the dependent variables should be compared with a placebo control group in order to assess the influence of important elements of the clinical context, such as the relationship with the therapist or the experience with the environment [22,43].

### 4.1. Limitations of the Study

This observational study without a control group primarily aimed to assess the safety of self-induced myofascial therapy using a foam roller in people with hemophilia. The study design is the main limitation for generalizing the results. Similarly, the absence of a placebo control group prevents evaluating the effectiveness of the intervention in improving secondary variables. The performance of clinical trials, taking into account the views of the subjects and considering their experience regarding the treatment, would be another measure of methodological quality to be considered in future studies.

The absence of an evaluation of changes in joint health or muscle activation and flexibility prevents determining the effect of this intervention on important clinical variables in the typical sequelae of hemophilic arthropathy. Failure to implement ultrasound control measures is another limitation for the measurement of the results regarding the frequency of hemarthrosis, given the possible development of subclinical bleeding.

### 4.2. Recommendations for Future Research

Randomized clinical studies are needed to confirm the results reported in this study. Likewise, methodological quality measures should be implemented, such as a follow-up period, sample randomization and intent-to-treat analysis. These randomized clinical trials should present between group differences and not report within-group differences as evidence of effectiveness. It would be useful to analyze any possible differences in the intake of anti-inflammatory drugs during the study period.

## 5. Conclusions

A self-induced fascial therapy protocol using a foam roller is safe for people with hemophilic ankle arthropathy. Changes in pain intensity, plantar flexion, and functionality have been observed in the patients included in the study. Multicenter, randomized clinical studies in patients with hemophilic ankle arthropathy are needed to confirm the safety and assess the effectiveness of self-induced fascial therapy.

## Figures and Tables

**Table 1:** Descriptive characteristics of the patients.

Variables		Median	Interquartile Range
Age (years)		36	9.00
Weight (kg)		84	21.50
Height (cm)		172	7.75
Ankle-joint health (0–20)		11	6.00
		**n**	**%**
Type of hemophilia	A	16	80
	B	4	20
Severity of hemophilia	Severe	18	90
	Moderate	2	10
Type of treatment	Prophylaxis	17	85
	On demand	3	15
Development of inhibitors	Yes	3	85
	No	17	15

**Table 2:** Changes in the frequency of hemarthrosis.

Events	Period	Number	Poisson Regression Analysis		Negative Binomial Regression Analysis	
			z (*p*)	SE (95%CI)	z (*p*)	SE (95%CI)
Hemarthrosis	Before T0 (56 days)	12	−1.39 (0.16)	0.50 (−1.67; 0.28)	−1.34 (0.18)	0.51 (−1.70; 0.32)
	T0–T1 (56 days)	2				
	T1–T2 (56 days)	4				
	Dispersion parameter estimate			Alpha = 0.18	χ^2^ = 0.09; *p* = 0.38	

T0–T1: outcome measures for baseline to post-treatment assessments; T1–T2: outcome measures for post-treatment to follow-up assessments; T0–T2: outcome measures for baseline to follow-up assessments. z: Wald test; SE: standard error; 95%CI: 95% confidence interval.

**Table 3:** Means (and standard deviations) of the secondary variables and within-subject interaction results for each of the dependent variables of the study.

Variables	T0	T1	T2	W	F	Sig.	η^2^_p_
Intensity of joint pain (0–10)	4.91 (2.15)	2.79 (1.97)	2.46 (2.09)	0.81	33.68	0.00 **	0.63
Dorsal flexion (degrees)	0.65 (8.72)	8.30 (19.31)	2.35 (7.11)	0.22	2.21	0.15	0.10
Plantar flexion (degrees)	36.05 (15.32)	41.50 (13.67)	40.30 (12.40)	0.93	5.22	0.01 *	0.21
Functionality (m)	173.06 (56.59)	184.85 (62.39)	178.39 (52.24)	0.87	5.34	0.01 *	0.22

Outcome measures at the baseline (T0), after the 8-week period of experimental intervention (T1), and after an additional 8-week follow-up (T2); W: Mauchly’s Sphericity Test, Sig.: significance. η^2^_p_: partial Eta-squared. Notes: * *p* < 0.01; ** *p* < 0.001

**Table 4:** Pairwise comparison analysis, mean difference [and 95% confidence interval] between the three assessments carried out.

Variables	T0–T1	T1–T2	T0–T2
Intensity of joint pain	2.11 [1.12; 3.10] **	0.33 [−0.34; 1.00]	2.44 [1.59; 3.29] **
Dorsal flexion	−7.65 [−19.97; 4.67]	5.95 [−5.76; 17.66]	−1.70 [−5.23; 1.83]
Plantar flexion	−5.45 [−10.66; −0.23] *	1.20 [−3.13; 5.53]	−4.25 [−8.60; 0.10]
Functionality	−11.78 [−19.42; −4.15] **	6.46 [−3.86; 16.78]	−5.31 [−15.55; 4.89]

T0–T1: outcome measures for baseline to post-treatment assessments; T1–T2: outcome measures for post-treatment to follow-up assessments; T0–T2: outcome measures for baseline to follow-up assessments. Notes: * *p* < 0.025; ** *p* < 0.001.

## References

[1] Gringeri A., Ewenstein B., Reininger A. (2014). The burden of bleeding in haemophilia: Is one bleed too many?. Haemophilia.

[2] Blanchette V.S., Key N.S., Ljung L.R., Manco-Johnson M.J., van den Berg H.M., Srivastava A. (2014). Definitions in hemophilia: Communication from the SSC of the ISTH. J. Thromb. Haemost..

[3] van Balen E.C., O’Mahony B., Cnossen M.H., Dolan G., Blanchette V.S., Fischer K., Gue D., O’Hara J., Iorio A., Jackson S. (2021). Patient-relevant health outcomes for hemophilia care: Development of an international standard outcomes set. Res. Pract. Thromb. Haemost..

[4] Rodriguez-Merchan E.C. (2012). Prevention of the musculoskeletal complications of hemophilia. Adv. Prev. Med..

[5] Fischer K., Poonnoose P., Dunn A.L., Babyn P., Manco-Johnson M.J., David J.A., van der Net J., Feldman B., Berger K., Carcao M. (2017). Choosing outcome assessment tools in haemophilia care and research: A multidisciplinary perspective. Haemophilia.

[6] Tonogai I., Sairyo K. (2020). A case of arthroscopic ankle arthrodesis for hemophilic arthropathy of the bilateral ankles. Int. J. Surg. Case Rep..

[7] Stephensen D., Bladen M., McLaughlin P. (2018). Recent advances in musculoskeletal physiotherapy for haemophilia. Ther. Adv. Hematol..

[8] Holstein K., Klamroth R., Richards M., Carvalho M., Pérez-Garrido R., Gringeri A. (2012). Pain management in patients with haemophilia: A European survey. Haemophilia.

[9] Cuesta-Barriuso R., Gómez-Conesa A., López-Pina J.A. (2014). Manual therapy in the treatment of ankle hemophilic arthropathy. A randomized pilot study. Physiother. Theory Pract..

[10] Cuesta-Barriuso R., Gómez-Conesa A., López-Pina J.A. (2014). Effectiveness of two modalities of physiotherapy in the treatment of haemophilic arthropathy of the ankle: A randomized pilot study. Haemophilia.

[11] Cuesta-Barriuso R., Trelles-Martínez R.O. (2018). Manual therapy in the treatment of patients with hemophilia B and inhibitor. BMC Musculoskelet. Disord..

[12] Pilat A. (2003). Inducción Miofascial. Aspectos Teóricos y Consideraciones Clínicas.

[13] Donoso-Úbeda E., Meroño-Gallut J., López-Pina J.A., Cuesta-Barriuso R. (2018). Safety and effectiveness of fascial therapy in adult patients with hemophilic arthropathy. A pilot study. Physiother. Theory Pract..

[14] Donoso-Úbeda E., Meroño-Gallut J., López-Pina J.A., Cuesta-Barriuso R. (2018). Safety of fascial therapy in adult patients with hemophilic arthropathy of ankle. A cohort study. Musculoskelet. Sci. Pract..

[15] Donoso-Úbeda E., Meroño-Gallut J., López-Pina J.A., Cuesta-Barriuso R. (2020). Effect of manual therapy in patients with hemophilia and ankle arthropathy: A randomized clinical trial. Clin. Rehabil..

[16] Pérez-Llanes R., Meroño-Gallut J., Donoso-Úbeda E., López-Pina J.A., Cuesta-Barriuso R. (2020). Safety and effectiveness of fascial therapy in the treatment of adult patients with hemophilic elbow arthropathy: A pilot study. Physiother. Theory Pract..

[17] Pérez-Llanes R., Donoso-Úbeda E., Meroño-Gallut J., López-Pina J.A., Cuesta-Barriuso R. (2020). Manual Therapy Effectively Decreases the Frequency of Joint Bleeding Improves Joint Health and Reduces Pain in Hemophilic Elbow Arthropathy: A Prospective Cohort Study. J. Rehabil. Med. Clin. Commun..

[18] Cuesta-Barriuso R., Pérez-Llanes R., Donoso-Úbeda E., López-Pina J.A., Meroño-Gallut J. (2021). Effects of myofascial release on frequency of joint bleedings, joint status, and joint pain in patients with hemophilic elbow arthropathy: A randomized, single-blind clinical trial. Medicine.

[19] MacDonald G.Z., Penney M.D., Mullaley M.E., Cuconato A.L., Drake C., Behm D.G., Button D.C. (2013). An acute bout of self-myofascial release increases range of motion without a subsequent decrease in muscle activation or force. J. Strength Cond. Res..

[20] Bialosky J.E., Beneciuk J.M., Bishop M.D., Coronado R.A., Penza C.W., Simon C.B., George S.Z. (2018). Unraveling the Mecha-nisms of Manual Therapy: Modeling an Approach. J. Orthop. Sports Phys. Ther..

[21] Geri T., Viceconti A., Minacci M., Testa M., Rossettini G. (2019). Manual therapy: Exploiting the role of human touch. Musculoskelet. Sci. Pract..

[22] Newell D., Lothe L.R., Raven T.J.L. (2017). Contextually Aided Recovery (CARe): A scientific theory for innate healing. Chiropr. Man. Therap..

[23] Hughes G.A., Ramer L.M. (2019). Duration of myofascial rolling for optimal recovery, range of motion, and performance: A systematic review of the literature. Int. J. Sports Phys. Ther..

[24] Ranbhor A.R., Prabhakar A.J., Eapen C. (2021). Immediate effect of foam roller on pain and ankle range of motion in patients with plantar fasciitis: A randomized controlled trial. Hong Kong Physiother. J..

[25] Wilke J., Vogt L., Banzer W. (2018). Immediate effects of self-myofascial release on latent trigger point sensitivity: A randomized, placebo-controlled trial. Biol. Sport.

[26] Warren A.J., LaCross Z., Volberding J.L., O’Brien M. (2020). Acute outcomes of myofascial decompression (cupping therapy) compared to self-myofascial release on hamstring pathology after a single treatment. Int. J. Sports Phys. Ther..

[27] Fischer K., De Kleijn P. (2013). Using the haemophilia joint health score for assessment of teenagers and young adults: Exploring reliability and validity. Haemophilia.

[28] Cuesta-Barriuso R., Donoso-Úbeda E., Meroño-Gallut J., Pérez-Llanes R., López-Pina J.A. (2020). Functionality and range of motion in patients with hemophilic ankle arthropathy treated with fascial therapy. A randomized clinical trial. Musculoskelet. Sci. Pract..

[29] Cuesta-Barrius R., Pérez-Llanes R., López-Pina J.A., Donoso-Úbeda E., Meroño-Gallut J. (2022). Manual therapy reduces the frequency of clinical hemarthrosis and improves range of motion and perceived disability in patients with hemophilic elbow arthropathy. A randomized, single-blind, clinical trial. Disabil. Rehabil..

[30] Hawksley H. (2000). Pain assessment using a visual analogue scale. Prof. Nurse Lond. Engl..

[31] Dworkin R.H., Turk D.C., Wyrwich K.W., Beaton D., Cleeland C.S., Farrar J.T., Haythornthwaite J.A., Jensen M.P., Kerns R.D., Ader D.N. (2008). Interpreting the clinical importance of treatment outcomes in chronic pain clinical trials: IMMPACT recommendations. J. Pain.

[32] Konor M.M., Morton S., Eckerson J.M., Grindstaff T.L. (2012). Reliability of three measures of ankle dorsiflexion range of motion. Int. J. Sports Phys. Ther..

[33] Tully E. (2005). The practical guide to range of motion assessment. Br. J. Sports Med..

[34] Connelly D., Thomas B., Cliffe S., Perry W., Smith R. (2009). Clinical utility of the 2-min walk test for older adults living in long-term care. Physiother. Can..

[35] Meroño-Gallut A.J., Cuesta-Barriuso R., Pérez-Llanes R., López-Pina J.A. (2020). Self-Myofascial Release Intervention and Mobile App in Patients with Hemophilic Ankle Arthropathy: Protocol for a Randomized Controlled Trial. JMIR Res. Protoc..

[36] Pallant J. (2013). SPSS Survival Manual.

[37] Meroño-Gallut J., Cuesta-Barriuso R. (2016). Design of a protocol myofascial therapy for the treatment of hemophilic arthropathy of the knee and ankle. Altern. Complement. Ther..

[38] Humphries T.J., Kessler C.M. (2015). Managing chronic pain in adults with haemophilia: Current status and call to action. Haemophilia.

[39] Bialosky J.E., Bishop M.D., Price D.D., Robinson M.E., George S.Z. (2009). The mechanisms of manual therapy in treating musculoskeletal pain: A comprehensive model. Man. Ther..

[40] Cavanaugh M.T., Döweling A., Young J.D., Quigley P.J., Hodgson D.D., Whitten J.H., Reid J.C., Aboodarda S.J., Behm D.G. (2017). An acute session of roller massage prolongs voluntary torque development and diminishes evoked pain. Eur. J. Appl. Physiol..

[41] Škarabot J., Beardsley C., Štirn I. (2015). Comparing the effects of self-myofascial release with static stretching on ankle range-of-motion in adolescent athletes. Int. J. Sports Phys. Ther..

[42] Pablos A., Ceca D., Jorda A., Rivera P., Colmena C., Elvira L., Martínez-Arnau F.M., Valles S.L. (2020). Protective Effects of Foam Rolling against Inflammation and Notexin Induced Muscle Damage in Rats. Int. J. Med. Sci..

[43] Rossettini G., Testa M. (2018). Manual therapy RCTs: Should we control placebo in placebo con-trol?. Eur. J. Phys. Rehabil. Med..

